# Supplementary material for: Light-Responsive Polymeric Micellar Nanoparticles with Enhanced Formulation Stability
Source: Polymers (Basel). 2021 Jan 26;13(3):377. doi: 10.3390/polym13030377 (PMC7866127; doi:10.3390/polym13030377)
Supplement: Supplementary file 1 [file polymers-13-00377-s001.pdf]

polymers

MDPI

Supporting Information

Light-Responsive Polymeric Micellar Nanoparticles with Enhanced Formulation Stability

Kyoung Nan Kim [1], Keun Sang Oh [2], Jiwook Shim [3], Isabel R. Schlaepfer [4], Sana D. Karam [5] and Jung-Jae Lee [1,6,*]

1 Department of Chemistry, University of Colorado Denver, Denver, CO 80204, USA; kyoung.kim@ucdenver.edu
2 TGel Bio, Co, LTD, Seoul 06185, Korea; oks5486@nate.com
3 Department of Biomedical Engineering, Rowan University, Glassboro, NJ 08028, USA; shimj@rowan.edu
4 Division of Medical Oncology, Anschutz Medical Campus, University of Colorado, Aurora, CO 80045, USA; isabel.schlaepfer@cuanschutz.edu
5 Department of Radiation Oncology, Anschutz Medical Campus, University of Colorado, Aurora, CO 80045, USA; sana.karam@cuanschutz.edu
6 Department of Bioengineering, University of Colorado Denver, Denver, CO 80204, USA
* Correspondence: Jungjae.Lee@ucdenver.edu; Tel.: +303-315-7671

Citation: Kim K.N.; Oh K.S.; Shim J.; Schlaepfer I.R.; Karam S.D.; Lee J.-J. Light Responsive Polymeric Micellar Nanoparticles with Enhanced Formulation Stability *Polymers* **2021**, *13*, 377. https://doi.org/10.3390/polym13030377

Received: 5 January
Accepted: 22 January
Published: 26 January

Publisher's Note: MDPI stays neutral with regard to jurisdictional claims in published maps and institutional affiliations.

Copyright: © 2021 by the authors. Licensee MDPI, Basel, Switzerland. This article is an open access article distributed under the terms and conditions of the Creative Commons Attribution (CC BY) license (http://creativecommons.org/licenses/by/4.0/).

Figure S1. Synthesis of PEG-PLL-DNQ and PEG-PLL-Oct, and the chemical structures of DTX and NR: **S-2**

Figure S2. Wolff Rearrangement of PEG-PLL-DNQ upon UV light irradiation: **S-3.**

Figure S3. DLS size distribution and TEM image of **DTX-NP$_{oct}$: S-4.**

Table S1. Measurement of Drug Loading Efficiency (LE) and Capacity (LC)**: S-5.**

Figure S4. DLS measurement of size change of **NR-NP$_{Oct}$** in BSA solution: **S-6.**

Figure S5. HUVECs Viability of Polymers (PEG-PLL-DNQ & PEG-PLL-Oct)**: S-7.**

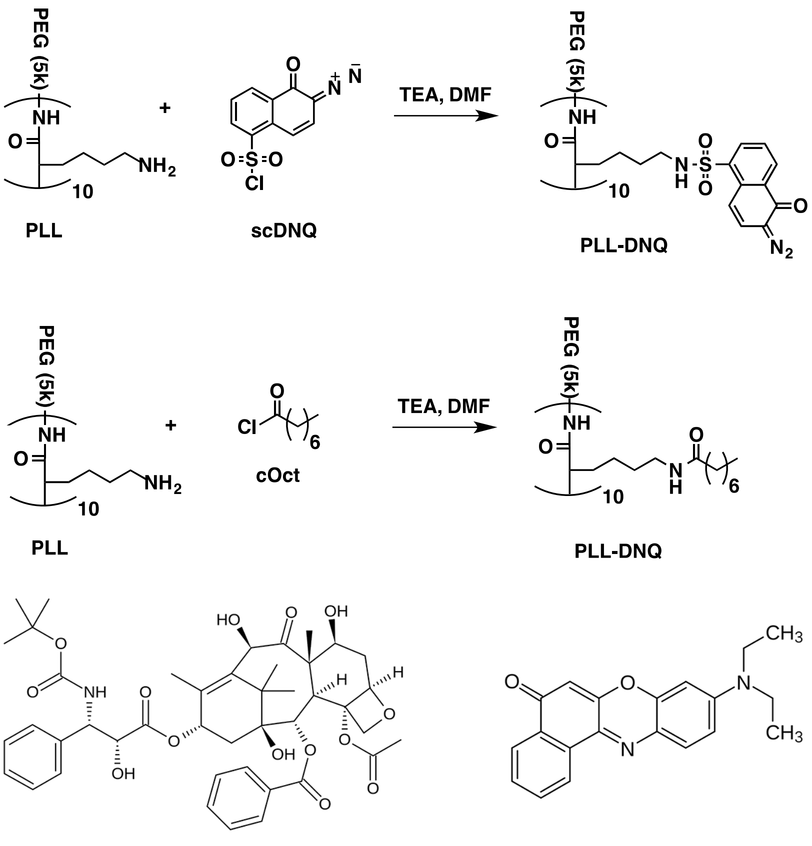

Decetaxel (DTX) **Nile Red (NR)**

Figure S1. Synthesis of PEG-PLL-DNQ and PEG-PLL-Oct, and the chemical structures of DTX and NR.

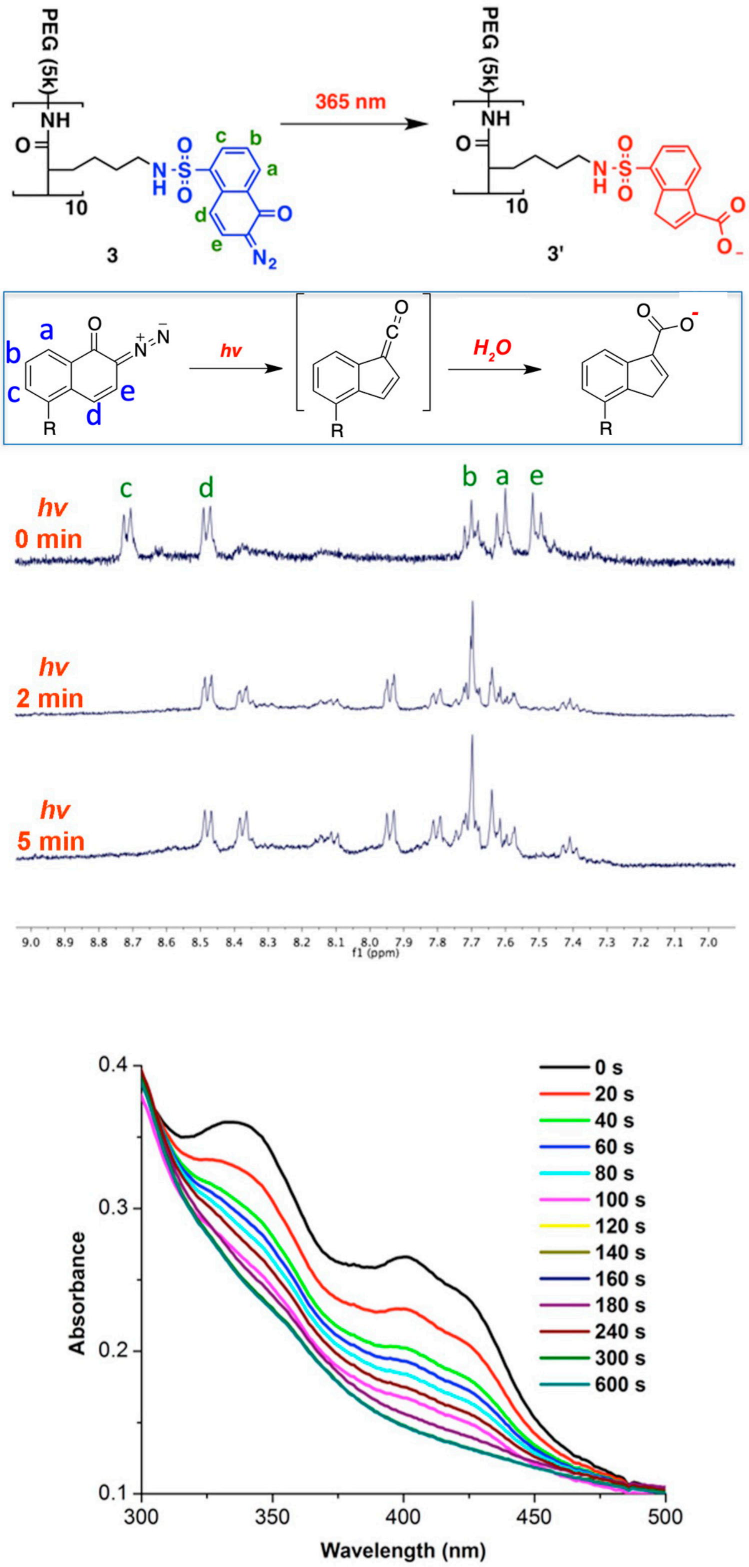

Figure S2. Wolff Rearrangement of PEG-PLL-DNQ upon UV light irradiation (365 nm, 50 mW/cm^2) (^{1}H NMR & UV/Vis Spectroscopy).

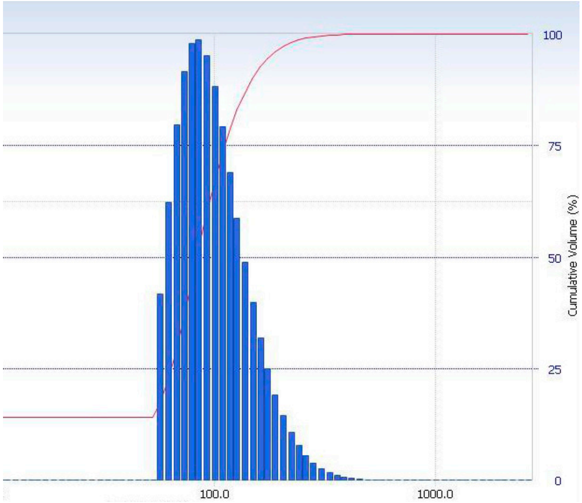

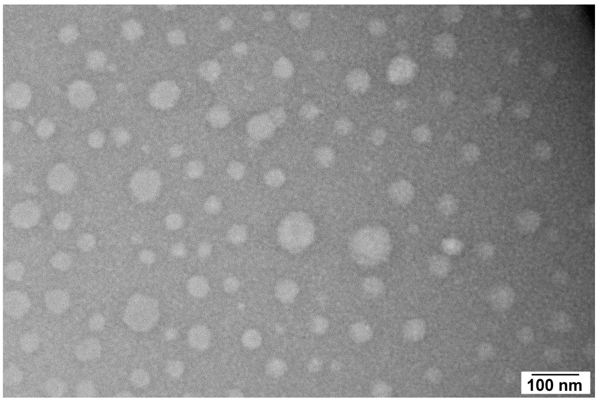

Figure S3. DLS size distribution and TEM image of **DTX-NP$_{oct}$**.

Table S1. Measurement of Drug Loading Efficiency (LE) and Capacity (LC).

Theoretical LD %	Actual LD % DTX-NP$_{DNQ}$	LD efficiency % DTX-NP$_{DNQ}$	Actual LD % DTX-NP$_{Oct}$	LD efficiency % DTX-NP$_{Oct}$
5	4.72 ± 0.07	94.4	4.43 ± 0.06	88.6
10	8.61 ± 0.16	86.1	7.91 ± 0.13	79.1
15	13.54 ± 0.27	90.3	8.73 ± 0.3	58.2
20	14.42 ± 0.49	72.1	9.20 ± 0.35	46.0

Determined by HPLC. Abbreviations: LD: loading. Data are mean ± SD (N = 4).

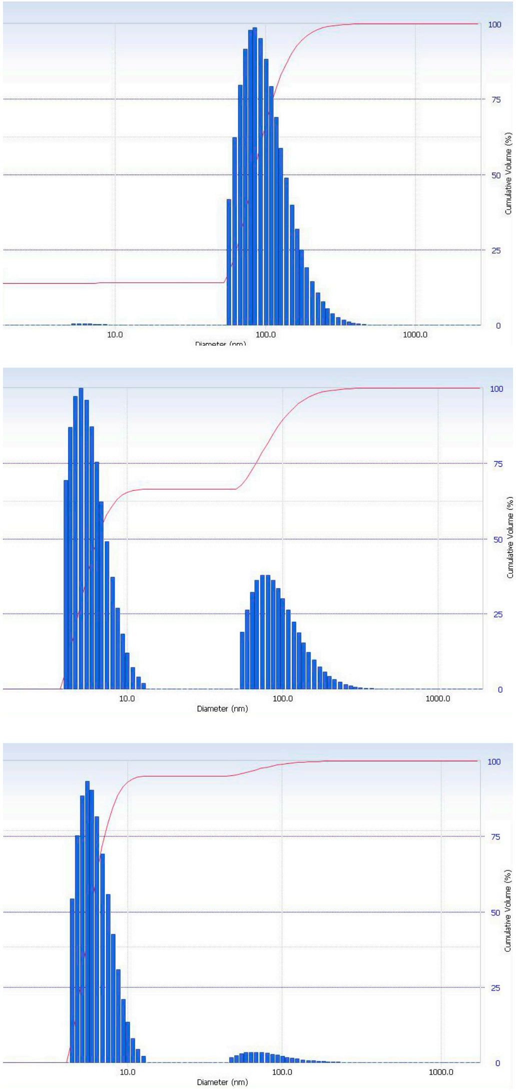

Figure S4. DLS measurement of size change of **NR-NP**$_{Oct}$ in BSA solution (0, 12, and 48 h).

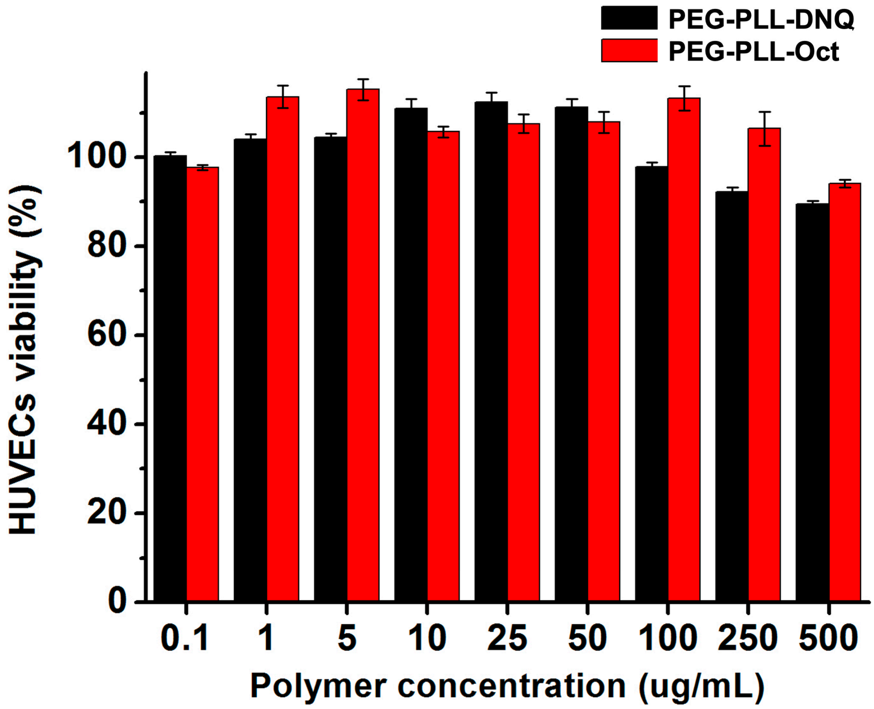

Figure S5. HUVECs Viability of Polymers (PEG-PLL-DNQ & PEG-PLL-Oct).
